# Efficacy of inhibition of IL-1 in patients with rheumatoid arthritis and type 2 diabetes mellitus: two case reports and review of the literature

**DOI:** 10.1186/s13256-015-0603-y

**Published:** 2015-06-02

**Authors:** Piero Ruscitti, Paola Cipriani, Luca Cantarini, Vasiliki Liakouli, Antonio Vitale, Francesco Carubbi, Onorina Berardicurti, Mauro Galeazzi, Marco Valenti, Roberto Giacomelli

**Affiliations:** 1 Department of Biotechnological and Applied Clinical Sciences, University of L’Aquila, Rheumatology Unit, delta 6 building, PO Box 67100, L’Aquila, Italy; 2 Research Center of Systemic Autoinflammatory Diseases and Behçet’s Disease Clinic, Department of Medical Sciences, Surgery and Neurosciences, University of Siena,Policlinico Santa Maria alle Scotte, Viale Mario Bracci, PO Box 53100, Siena, Italy; 3 Department of Biotechnological and Applied Clinical Sciences, University of L’Aquila, Medical Statistic Unit, delta 6 building, PO Box 67100, L’Aquila, Italy

**Keywords:** Rheumatoid arthritis, Type 2 diabetes mellitus, Interleukin-1β, Anakinra

## Abstract

**Introduction:**

Rheumatoid arthritis is an autoimmune arthritis in which two inflammatory cytokines, tumor necrosis factor-α and interleukin-1β, play a critical role in the induction and progression of the disease. Several reports and data from registries have discussed the association between chronic inflammatory diseases and disorders in intermediary metabolism, pointing out that prevalence of peripheral insulin resistance and type 2 diabetes mellitus is increased among patients with rheumatoid arthritis. In addition, several studies have shown that type 2 diabetes mellitus may be considered an interleukin-1β inflammatory-mediated process, and both preclinical and clinical observations have reported the usefulness of interleukin-1 antagonism therapy in this disease.

**Case presentation:**

We describe the case of a 58-year-old Caucasian woman and a 74-year-old Caucasian man with rheumatoid arthritis associated with type 2 diabetes mellitus. In these patients, the inhibition of interleukin-1β not only induced remission for rheumatoid arthritis, but successfully controlled their metabolic status.

**Conclusions:**

We report the positive effects of the inhibition of interleukin-1 in two patients with rheumatoid arthritis associated with type 2 diabetes mellitus, with both reaching the therapeutic targets of their diseases by using a single biological agent and tapering or discontinuing their antidiabetic therapies. These findings suggest that targeting interleukin-1 might be considered a good therapeutic option for the treatment of rheumatoid arthritis associated with type 2 diabetes mellitus.

## Introduction

Interleukin-1β (IL-1β) and tumor necrosis factor-α (TNF-α) play a critical role in the induction and progression of rheumatoid arthritis (RA), and the efficacy of therapies targeting these two inflammatory cytokines confirms their prominent role in the disease [1]. Both several reports and data from registries have suggested that the prevalence of type 2 diabetes mellitus (T2DM) is increased in patients with RA [2,3]. In addition, several studies have shown that T2DM may be considered an IL-1β inflammatory-mediated process, and both preclinical and clinical observations have reported the usefulness of IL-1 antagonism therapy in this disease [4-6]. In this paper, we describe two patients with RA associated with T2DM, in which the inhibition of IL-1 induced remission for RA and successfully controlled the metabolic status, suggesting that targeting IL-1 might be considered a good therapeutic option for the treatment of RA associated with T2DM.

## Case presentation

### Case report one

A 58-year-old Caucasian woman was referred to our clinic due to the insidious onset, in previous months, of arthralgias and arthritis. Her symptoms involved wrists, hands, shoulders and knees. She also reported notable morning stiffness and fatigue, limiting her daily activities. The results of her cardiological, pulmonary, dermatological, abdominal and neurological examinations were unremarkable. In her medical history, both essential hypertension and osteoporosis were reported. Laboratory findings showed an increase of inflammatory markers and were negative for rheumatoid factor (RF), antinuclear antibodies and anti-cyclic citrullinated peptide (ACPA). A radiological examination of her wrists and hands showed multiple bone erosions and juxta-articular osteoporosis. Her Disease Activity Score in 28 Joints (DAS28), Simple Disease Activity Index (SDAI) score and Patient Global Visual Analogue Scale (PG-VAS) score were 4.5, 28 and 67mm, respectively, and active seronegative RA was diagnosed. Combination therapy with methotrexate (15mg/weekly) (TEVA, Israel), hydroxychloroquine (400mg/daily) (Sanofi, Italy) and a low dose of prednisone (10mg/daily) (Bruno Farmaceutici, Italy) was prescribed. She experienced a long clinical remission (her DAS28, SDAI and PG-VAS scores were 2.2, 8.2 and 25mm, respectively, after one year).

She experienced a new disease flare-up after two years, characterized by multiple arthritis (wrists, hands and knees) and an increase of morning stiffness and fatigue (her DAS28, SDAI and PG-VAS scores were 5.2, 34.4 and 88mm, respectively). Infliximab (400mg/bi-monthly) was introduced into her treatment regimen and a new clinical remission was reached (her DAS28, SDAI and PG-VAS scores were 2.4, 6.8 and 30mm, respectively) for seven years. At this time, after her diagnosis of T2DM (fasting plasma glucose (FPG) level 189mg/dL, glycated hemoglobin (HbA1c) level 62mmol/mol, 7.8% (4.8-5.9%)), she began taking a new oral hypoglycemic drug (metformin 500mg trice/daily). One year later, following a new flare-up of her disease involving arthritis of her wrists, hands, elbows, shoulders and knees (her DAS28, SDAI and PG-VAS scores were 6.27, 34.6 and 80mm, respectively), her anti-TNF-α treatment with infliximab (MSD, USA) was discontinued. Both her methotrexate and steroids dosage remained stable; the hydrossicloroquine was discontinued due to poor compliance. Anakinra (Sobi, Sweden), a recombinant form of a human IL-1 receptor antagonist, was introduced into her therapy regimen (100mg/daily).

**Figure 1:**
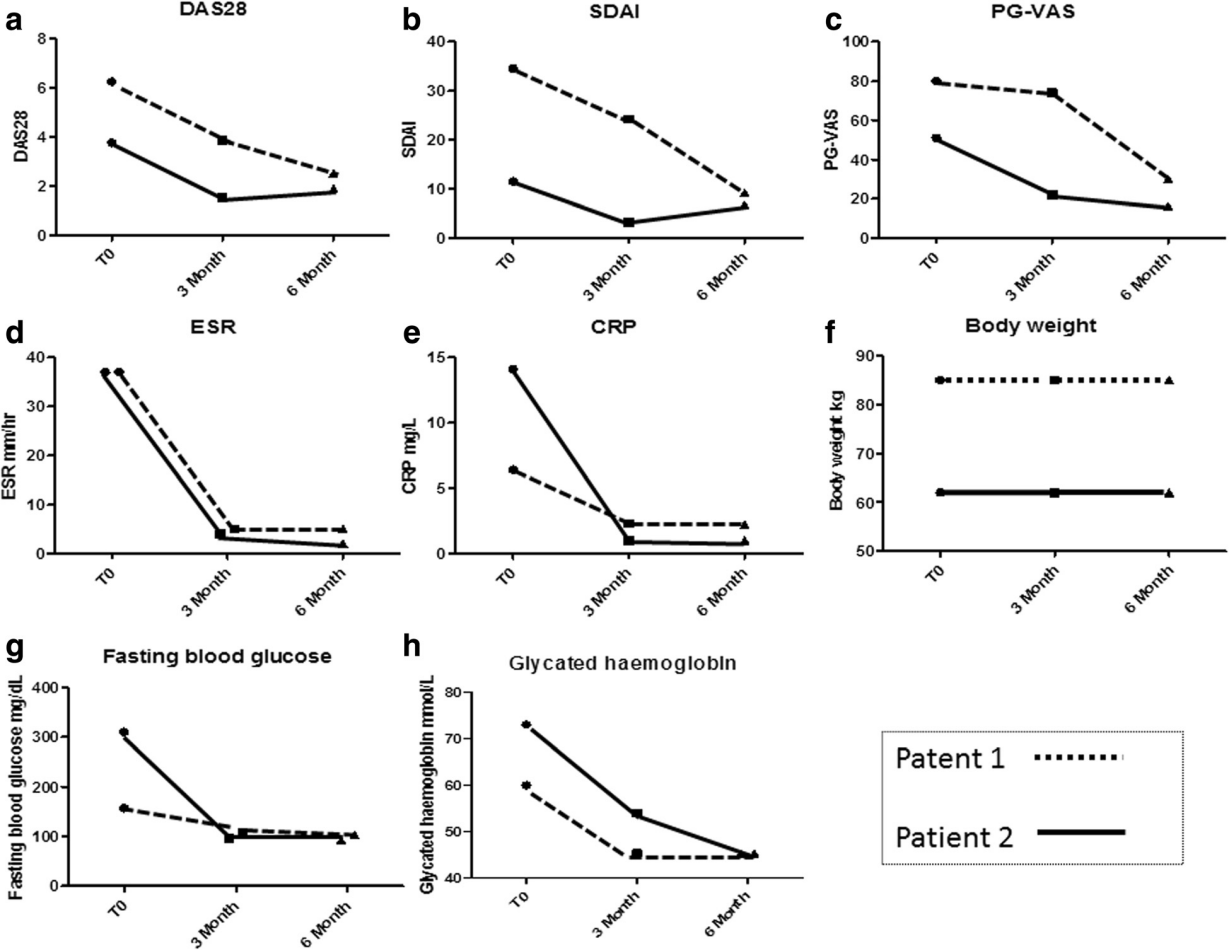
Clinical charateristics of evaluated patients during six months of follow-up. Disease Activity Score in 28 Joints (DAS28) **(a)**, Simple Disease Activity Index (SDAI) **(b)**, Patient Global Visual Analogue Scale (PG-VAS) **(c)**, erythrocyte sedimentation rate (ESR) **(d)**, C-reactive protein values (CRP) **(e)**, body weight **(f)**, fasting plasma glucose **(g)** and glycosylated hemoglobin **(h)** concentrations in our two patients at the beginning of anakinra treatment and after three and six months of follow-up.

With regards to therapy for her T2DM, a stable dosage of metformin was continued (FPG level 127mg/dL, HbA1c level 60mmol/mol, 7.6%). In the six subsequent months a new clinical remission was observed. After three months of therapy, her DAS28, SDAI and PG-VAS scores were 3.88, 24.2 and 74mm, respectively. At the same time, her FPG and HbA1c levels were 108mg/dL and 46mmol/mol, 6.3%, respectively. After six months of therapy, her DAS28, SDAI and PG-VAS scores were 2.52, 9.2 and 30mm, respectively. Furthermore, repeated tests showed a stable further reduction in her FPG and HbA1c levels at 103mg/dL and 46mmol/mol (6.3%) (4.8-5.9%), respectively. In Figure 1, the values of DAS28, SDAI, PG-VAS, inflammatory markers, FPG and HbA1c are reported. A reduction of daily intake of metformin was observed (metformin 500mg once/daily). Fasting insulin levels were increased following treatment with anakinra: 34pmoles/liter at baseline; 43 pmoles/liter at three months and 69pmoles/liter at six months, respectively. Similarly, we observed that fasting C-peptide levels were increased: 0.4nmoles/liter at baseline; 0.6nmoles/liter at three months and 0.8nmoles/liter at six months, respectively. During the follow-up period, her weight, frequency of physical exercise and diet/caloric intake did not modify. No side effects were observed.

### Case report two

A 74-year-old Caucasian man with an eight-year history of RA, treated by a combination therapy with methotrexate (15mg/weekly), hydroxychloroquine (200mg/daily) and methylprednisolone (Pfizer, Italy) (16mg/daily at the beginning, progressively tapered to 3 to 4mg/daily), obtaining a stable and sustained good clinical response (DAS28 not available) was admitted to our unit with a new flare-up of the disease consisting of multiple swelling joints, mainly involving his hands and feet, with a symmetrical pattern. He showed DAS28, SDAI and PG-VAS scores of 3.78, 11.5 and 51mm, respectively. In addition, screening for autoimmunity highlighted positivity for RF (100UI/mL) and ACPA (1326UI/mL). He also reported a history of uncontrolled T2DM in the last 12 months, treated with repaglinide 0.5mg twice daily (Novo Nordisk Farmaceutici, Italy) (FPG level 310mg/dL, HbA1c level 73mmol/mol, 8.8% (4.8-5.9%)) despite the lower levels of methylprednisolone (3 to 4mg/daily) (Pfizer, Italy) employed. Anakinra (100mg/daily subcutaneously) (Sobi, Sweden) was started, in addition to methotrexate (10mg/weekly) (TEVA, Israel) and methylprednisolone (3-4mg daily) (Pfizer, Italy), leading to clinical improvement within a few days.

At his three-month follow-up, his DAS28, SDAI and PG-VAS scores had dropped to 1.53, 3.3 and 22mm, respectively. In addition, although oral anti-diabetic therapy was tapered off eight days after anakinra introduction due to a poor compliance, an improvement in glycemic control was noted (his FPG and HbA1c levels were 96mg/dL and 54mmol/mol, 7.1% (4.8-5.9%), respectively). At six months his DAS28, SDAI and PG-VAS scores were 1.89, 6.7 and 20mm, respectively, and a further improvement of both his FPG and HbA1c levels was observed (92mg/dL and 45.36mmol/mol, 6.3% (4.8-5.9%), respectively). His fasting insulin level was increased following treatment with anakinra: 36pmoles/liter at baseline, 53pmoles/liter at three months and 77pmoles/liter at six months, respectively. Similarly, we observed that his fasting C-peptide levels were increased: 0.6nmoles/liter at baseline, 1.1nmoles/liter at three months and 1.5nmoles/liter at six months, respectively. His weight, frequency of physical exercise and diet/caloric intake did not modify during the follow-up. No side effects were observed.

## Discussion

We show that anakinra, prescribed for the treatment of relapsing RA in two rheumatic patients with associated T2DM, controls not only the clinical pictures of RA, but also their metabolic status, confirming previous reports suggesting that IL-1 blockade may be helpful in patients with T2DM and inflammatory rheumatic diseases [7].

In both case studies, our patients reached a sustained DAS28 remission after introduction of anakinra, without any side effects observed during the follow-up periods. IL-1β is highly expressed in RA, promoting activation of leukocytes, endothelial cells, chondrocytes and osteoclasts [1], and targeting IL-1 reduces inflammation, joint damage and slows the radiographic progression in patients with RA [8]. Moreover, no opportunistic infections in treated patients have been reported [8].

Diabetes mellitus is a chronic disease due to a failure in both producing and using insulin. When compared with healthy people, patients with T2DM display a two-four-fold increased risk of coronary artery disease [2,3]. On the other hand, it is well known that RA is an independent risk for cardiovascular disease (CVD), and at present, cardiovascular (CV) events are the leading cause of death in patients with RA [2,3,9]. A prospective longitudinal large cohort study showed that traditional CV risk factors and markers of RA severity might both contribute in predicting fatal CV events [10]. On this basis, our patients (with both RA and T2DM) displayed a much higher risk of CVD and related mortality.

In our patients, we observed that anakinra administered to treat RA controlled their metabolic disorder. A significant reduction of both FPG and HbA1c levels was observed. It is well known that the pharmacologic interventions for T2DM are aimed at improving both FPG and HbA1c levels, as suggested by the American Diabetes Association and the American Association of Clinical Endocrinologists [11]. Large clinical trials have shown that intensive treatments are associated with a significant decrease of CV events [5,11]. It must be pointed out that by treating our patients with an anti-IL-1 agent, we were able to reach the optimal therapeutic targets for both RA and T2DM, thus decreasing their CVD risk according to the European League Against Rheumatism recommendations [9]. These results may suggest new therapeutic possibilities in patients with both RA and T2DM.

It must be pointed out that when diabetic patients are treated with oral antidiabetic drug monotherapy, after an initial improvement, a progressive and inexorable decline in β-cell function may be observed. Many studies showed that after three years of treatment, the disease may be controlled by monotherapy in only 50%, and in only 25% after nine years [11], confirming that T2DM is a progressive disease and combined therapies are needed to achieve and maintain metabolic targets [4,5,11]. In our study, one patient decreased her daily antidiabetic drug intake, while the other patient discontinued the specific antidiabetic therapy. In this setting, anakinra was able to determine both a stable DAS28 remission and a glycemic control after six months and that an anti-IL-1 therapy allowed our patients to control their rheumatic disease without increasing steroid treatment, associated with an increased risk of CV events, in a dose-related manner [12].

Taken together, these results support that IL-1 antagonism may be a specific therapy in RA with associated T2DM. Although the role of IL-1β has been extensively studied in RA, recently several studies have suggested this cytokine as a key cytokine in the pathogenesis of T2DM [4,5]. In fact, under the influence of higher glycemic levels, islets macrophages start to produce inflammatory cytokines, including IL-1β and TNF-α, which increase the β-cell apoptosis rate, thus contributing to the impairment in pancreatic secretory function [4,5].

In this setting, Larsen *et al*. performed a double blind, parallel-group trial, involving 70 T2DM patients randomly assigned to receive 100mg of anakinra or placebo. At 13 weeks, the anakinra group showed an improvement of HbA1c levels, the C-peptide secretion was enhanced and a reduction in the ratio of proinsulin to insulin and in levels of IL-6 and C-reactive protein (CRP) was observed [6]. Furthermore, the extension of this study showed that this improvement lasted at least 39 weeks after treatment withdrawal [13]. In our patients, we observed a reduction of HbA1c and CRP levels associated with an increase of insulin levels and C-peptide, mirroring what was already observed in the study by Larsen *et al*., and suggesting an improvement of β-cell function. In this context, it has been recently shown that anakinra is able to increase insulin secretion [14], and this insulin availability may explain its metabolic effects.

Recently, Moran *et al*. reported that anakinra therapy was not effective in type 1 diabetes (T1DM) patients [15]. It must be pointed out that the insulitis in T1DM is mainly driven by an autoimmune-mediated process rather than an autoinflammatory process as observed in T2DM, and this different mechanism suggests that IL-1 blockade may not be considered a good strategy to inhibit the pathogenesis of T1DM [5,15].

## Conclusions

In conclusion, we report the positive effects of anakinra for two patients with RA associated with T2DM reaching the therapeutic targets of both the diseases, using a single biological agent, and allowing us to taper or discontinue their antidiabetic therapies. In this setting, further specifically designed studies must be performed to evaluate the safety and long-term efficacy of anakinra in patients with RA associated with T2DM, and the subsequent reduction of CV events in this population. On these bases, an open, randomized, controlled, double-arm, multi-center study, whose primary endpoint is the efficacy of anakinra in controlling the signs and symptoms of T2DM in patients with RA affected by this metabolic disorder, is actually ongoing in our country (TRACK study, Clincaltrials.gov identifier: NCT02236481).

## Consent

Written informed consent was obtained from the patients for publication of this case report. A copy of the written consent is available for review by the Editor-in-Chief of this journal.

## Abbreviations

ACPA: Anti-cyclic citrullinated peptide
CRP: C-reactive protein
CV: Cardiovascular
CVD: Cardiovascular disease
DAS28: Disease activity score in 28 joints
FPG: Fasting plasma glucose
HbA1c: Glycated hemoglobin
IL-1β: Interleukin-1β
PG-VAS: Patient global visual analogue scale
RA: Rheumatoid arthritis
RF: Rheumatoid factor
SDAI: Simple disease activity index
T1DM: Type 1 diabetes
T2DM: Type 2 diabetes mellitus
TNF-α: Tumor necrosis factor-α.
